# *De Novo* Assembly and Transcriptome Characterization of Canine Retina Using High-Throughput Sequencing

**DOI:** 10.1155/2015/638679

**Published:** 2015-12-16

**Authors:** Bhaskar Reddy, Amrutlal K. Patel, Krishna M. Singh, Deepak B. Patil, Pinesh V. Parikh, Divyesh N. Kelawala, Prakash G. Koringa, Vaibhav D. Bhatt, Mandava V. Rao, Chaitanya G. Joshi

**Affiliations:** ^1^Ome Research Facility, Department of Animal Biotechnology, Anand Agricultural University, Anand, Gujarat 388001, India; ^2^Department of Zoology, Genetic Diagnostic Centre, University School of Sciences, Gujarat University, Ahmedabad, Gujarat 380009, India; ^3^Datar Genetics Ltd., F-8 D Road, Ambad, Nasik, Maharashtra 422010, India; ^4^Department of Veterinary Surgery & Radiology, College of Veterinary Science & Animal Husbandry, Anand Agricultural University, Anand, Gujarat 388001, India; ^5^Department of Pharmaceutical Science, Saurashtra University, Rajkot, Gujarat 360005, India

## Abstract

We performed transcriptome sequencing of canine retinal tissue by 454 GS-FLX and Ion Torrent PGM platforms. RNA-Seq analysis by CLC Genomics Workbench mapped expression of 10,360 genes. Gene ontology analysis of retinal transcriptome revealed abundance of transcripts known to be involved in vision associated processes. The* de novo* assembly of the sequences using CAP3 generated 29,683 contigs with mean length of 560.9 and N50 of 619 bases. Further analysis of contigs predicted 3,827 full-length cDNAs and 29,481 (99%) open reading frames (ORFs). In addition, 3,782 contigs were assigned to 316 KEGG pathways which included melanogenesis, phototransduction, and retinol metabolism with 33, 15, and 11 contigs, respectively. Among the identified microsatellites, dinucleotide repeats were 68.84%, followed by trinucleotides, tetranucleotides, pentanucleotides, and hexanucleotides in proportions of 25.76, 9.40, 2.52, and 0.96%, respectively. This study will serve as a valuable resource for understanding the biology and function of canine retina.

## 1. Introduction

The retina is composed of a neural cell layer and a retinal pigment epithelial cell layer. Two types of photoreceptor cells, rods and cones, in the neural cell layer convert light signals to changes in membrane potential, organized through complex layers of the neural cells and transmitted to the brain through the fibers of the optic nerve [1, 2]. The retinal pigment epithelium (RPE) plays an important role in supporting the function of the photoreceptor cells and serves as a blood-retina barrier [3]. Photoreceptor cells and the RPE are of interest physiologically as well as pathologically in relation to retinal degeneration [4–7]. Furthermore, photoreceptor cells serve as a model for the investigation of the development and differentiation of neural cells [8] and its normal function is dependent upon each cell type working properly in a coordinated fashion. Multiple disorders, that is, diabetes, age-related macular degeneration, inherited retinal degeneration (IRD), cancer, and so forth, affect the retina and cause vision loss at all ages [9].

Massively parallel, high-throughput sequencing platforms have provided possibility for genome-wide observations of the transcriptional makeup of retina genes. The transcriptome is the complete set of transcripts in a cell at a specific stage or under given physiological condition [10]. Understanding cell development, physiology, and disease may be improved by genome-wide characterization of the retinal transcriptome [11, 12]. High-throughput mRNA sequencing allows simultaneous transcript discovery and abundance estimation [13]. High-throughput sequencing data have a wide dynamic range of transcript expression for quantification and identification of rare transcripts within the constraints of the depth of coverage. With microarray technologies, transcripts can only be detected based on prior knowledge required for probe placement [14]. The size and structure of transcripts can be accurately measured by RNA-Seq, as compared to array hybridization, which does not provide any information on transcript size and splice variation. The microarray based gene expression of canine has been reported for lungs, brain, heart, kidney, liver, lymph node, pancreas, skeletal muscle, and spleen tissues [15]. The characterization of causative mutations for retinal blindness disorders has been of limited success due to poor availability of information on gene expression and underlying molecular mechanisms that trigger degenerative processes. To the best of our knowledge, there are no reports on transcriptome profiling of retinal tissue of dog. Hence, the present study was undertaken with the objective of developing a catalog of genes expressed in canine retina and their functional annotation.

## 2. Materials and Methods

### 2.1. Tissue Collection

The retinal tissues of both eyes were taken from a female nondescript dog, approximately 4-5 years old, that had an automobile accident and succumbed during the treatment at the Department of Veterinary Surgery & Radiology, AAU, Anand, Gujarat, India. Tissues were washed with sterile phosphate buffer saline solution, transferred immediately in the “RNA later,” and stored in liquid nitrogen for downstream processing.

### 2.2. mRNA Extraction, Library Preparation, and Sequencing

Total RNA was extracted from 100 mg of both tissues using TRIzol (Invitrogen Life Technologies, CA) reagent as per the manufacturer's instructions. DNase treatment was given to remove the DNA contamination. The quantity and quality of RNA were evaluated using NanoDrop1000 spectrophotometer (Thermo Fisher Scientific) as well as Bioanalyzer 2100 (Agilent Technologies, CA). Total mRNA was isolated from total RNA sample using mRNA isolation kit (Roche Diagnostics, Switzerland) as described in the manufacturer's protocols. Total isolated mRNA was again quality checked on Bioanalyzer 2100 using RNA 6000 nano Chip kit (Agilent Technologies, CA). cDNA and library preparations were carried out using kits of 454 GS-FLX sequencing and Ion Torrent mRNA library preparation. Both platforms based cDNA libraries were sequenced on 454 GS-FLX and Ion Torrent PGM sequencers as per the manufacturer's instructions. The brief steps for sequencing are mRNA fragmentation, adapter ligation, cDNA preparation, emulsion PCR based library amplification, and library enrichment.

### 2.3. Read Mapping and Gene Expression Analysis

The generated reads of both datasets were pooled and subjected to quality screening using PRINSEQ. Reads with less than 60 bases of read length, with mean read quality of less than 20, and with duplicate reads were removed [16]. The quality screened data was processed for the mapping and gene expression analysis. Reads were mapped to canine annotated genome assemblies using CLC Genomics Workbench 4.9 software. To quantify gene expression, the RNA-Seq analysis tool was used as previously described [17] allowing for no more than 2 mismatches per read. The annotated genome was downloaded from NCBI for canine genome build CanFam3.1.

### 2.4. Functional Annotation of Transcripts

The genes that were expressed with RPKM (reads per kilobase of exon model per million mapped reads) value of ≥0.5 were taken for functional annotation. Genes which were expressed with RPKM < 0.5 were excluded from the functional annotation. The genes (gene ontology) were annotated using Database for Annotation, Visualization and Integrated Discovery (DAVID) Version 6.7 [18] and analyzed for gene enrichment using Functional Annotation Tool. Genes associated with retinal function were retrieved from RetNet retinal information network database (https://sph.uth.edu/retnet/home.htm).

### 2.5. Sequence Data Processing and* De Novo* Assembly

The downstream analysis was carried out from total data obtained from 454 GS-FLX and Ion Torrent. The duplicate reads, chimeric reads, minimum length (<30 bp), quality mean read length (<20), and end trimming were performed using PRINSEQ tool [16]. Quality screened data was then processed for assembly. Reads were assembled using contig assembly program-3 (CAP3) [19] with default parameters (overlap similarity score cutoff 90, overlap percent identity cutoff 90, mismatch score (−5), and base quality cutoff 20).

### 2.6. Comparative Annotation of Assembled Contigs

The annotations of contigs were carried out by BLASTx searches of all contigs with reference to the NCBI nonredundant (nr) database using Blast2GO [13] with *e*-value cutoff of 1*∗*10^−6^. The assembled sequences were subjected to KEGG pathways assignment using the online KEGG [20] Automatic Annotation Server (KAAS) (http://www.genome.jp/kegg/kaas/) Ver. 1.67x with default parameters. KEGG pathway analyses of contigs were performed on KASS server using Bidirectional Best Hit.

### 2.7. Full-Length cDNA Prediction

All assembled transcripts (using CAP3) were submitted to in-house local BLASTx against protein sequence database of* Canis lupus familiaris* with *e*-value cutoff of 10^−5^ for identification of full-length cDNA. Prediction of full-length cDNAs was identified using online tool Target Identifier [21] with *e*-value cutoff of 10^−5^. The cDNA sequence was recognized as a full-length cDNA only if it has the start codon (ATG) and poly(A) tail codon.

### 2.8. ORF Identification

The assembled contigs were uploaded to online tool ORFPredictor [22] to identify open reading frames in the assembled contigs with an *e*-value cutoff of 10^−5^.

### 2.9. Identification of SSR/Microsatellites and Repeat Elements

The SSRs were identified from assembled sequences using SSR Locator [23] with threshold of 6 for di- and 5 for tri-, tetra-, penta-, and hexanucleotide repeats.

## 3. Results

### 3.1. Sequencing Analysis and Mapping Statistics

RNA sequencing carried out on Ion Torrent yielded a total of 569,066 quality reads with mean read length of 145.79 bp and on 454 GS-FLX yielded a total of 231,088 quality reads with mean read length of 373.19 bp. Out of 226,684 counted fragments, 222,296 reads mapped uniquely whereas 4,388 reads mapped nonspecifically with reference genome (Table 1). A total of 10,360 genes were identified out of 28,455 reference genes of canine transcriptome. The annotated reference assembly was downloaded from NCBI Genome browser CanFam3.1 reference genome (ftp://ftp.ncbi.nlm.nih.gov/genomes/Canis_lupus_familiaris/).

Among the reads mapping uniquely to protein coding genes, 34.05% located within exon reads and 14.63% on exon-exon reads. Nearly 48.00% located within the introns and 2.75% in the exon-intron reads (Table 2). The relatively high proportion of reads assigned to introns is not uncommon when the sequencing library preparation includes random priming of the mRNA [24].

### 3.2. Analysis of Gene Expression Profile of Retina

In our transcriptome study, 10,360 genes were expressed with RPKM values ≥ 0.50. In order to categorize the genes with different level of expression, genes were categorized based on RPKM values into three groups, that is, high (≥200 RPKM), medium (≥10–200 RPKM), and low (≥0.4–10 RPKM) expressed genes. There were 36 (0.78%) highly expressed genes, 1,850 (40.14%) moderately expressed genes, and 2,723 (59.08%) low expressed genes in the retina.

### 3.3. Functional Annotation of Retina Expressed Genes (GO Analysis)

The functional annotations of genes expressed in retinal tissue were performed using DAVID 6.7 web based annotation tool [18] which provides dynamic, controlled vocabulary and hierarchical relationships for the gene products in three categories: biological process, molecular function, and cellular component. Gene enrichment of GO terms was significant (*p* value < 0.01) in biological pathway, molecular function, and cellular component. The biological process was enriched in a total of 28 GO terms, cellular component in 34 GO terms, and molecular function in 27 GO terms.

#### 3.3.1. GO: Biological Process

**Figure 1:**
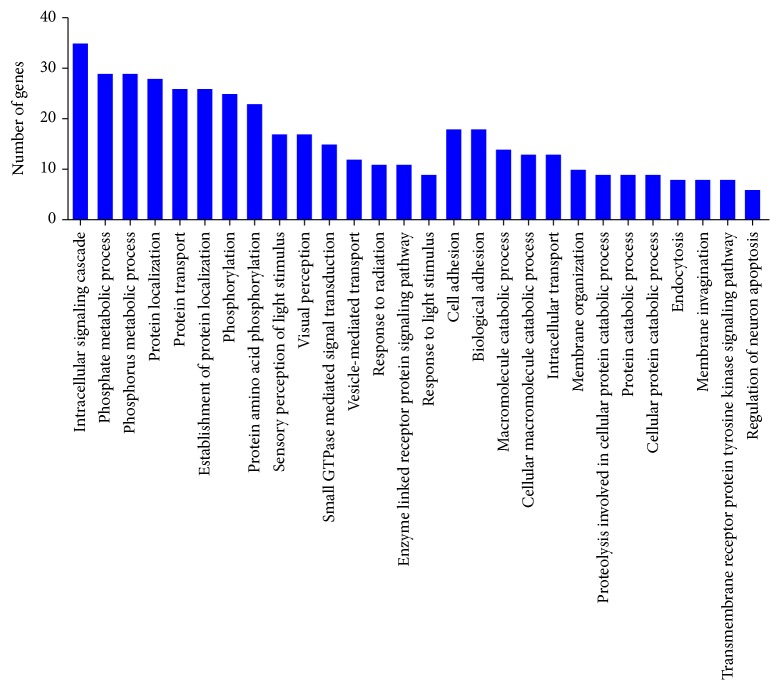
Functional annotation of expressed genes of retinal tissue in GO term: biological process.

The enrichment of expressed genes in biological process was observed for 316 genes in 28 GO terms which ranged between 25 and 6 genes. The assignment of GO terms (*p* value < 0.01) included intracellular signaling cascade (35 genes), phosphate metabolic process and phosphorus metabolic process (29 genes), protein localization (28 genes), protein transport and establishment of protein localization (26 genes), phosphorylation (25 genes), protein amino acid phosphorylation (23 genes), sensory perception of light stimulus and visual perception (17 genes), small GTPase mediated signal transduction (15 genes), macromolecule catabolic process (14 genes), response to radiation and enzyme linked receptor protein signaling pathway (11 genes), and response to light stimulus (9 genes). However, cell adhesion and biological adhesion (18 genes), macromolecule catabolic process (14 genes), cellular macromolecule catabolic process (13 genes), intracellular transport (13 genes), and membrane organization (10 genes) were enriched with *p* value ≤ 0.05 (Figure 1, Additional File S1, Sheet 1, in Supplementary Material available online at http://dx.doi.org/10.1155/2015/638679). The enrichment of genes in GO terms, namely, sensory perception of light stimulus, visual perception, response to light stimulus, response to radiation, cell adhesion, and biological adhesion, was consistent with retinal transcriptome of aged human and rat [25, 26].

#### 3.3.2. GO: Cellular Component

**Figure 2:**
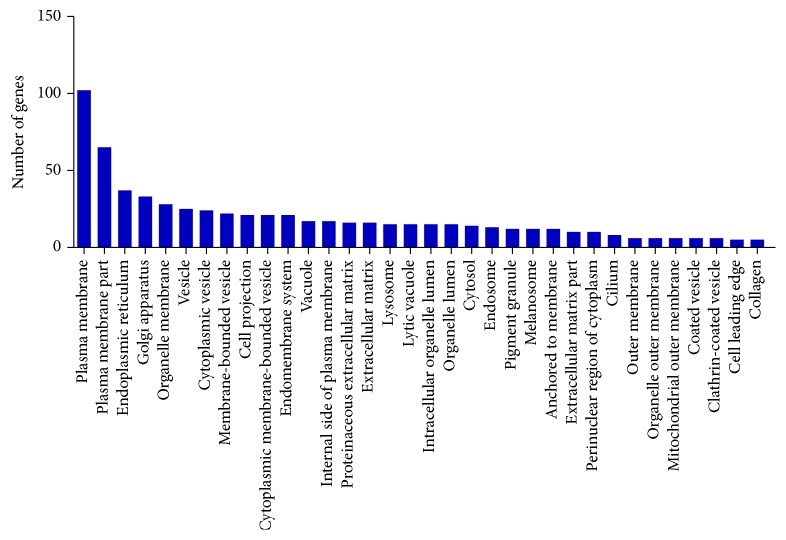
Functional annotation of expressed genes of retinal tissue in GO term: cellular component.

The enrichment of expressed genes in cellular component was observed for 293 genes which ranged between 102 and 5 genes in 34 GO terms (*p* value < 0.01), namely, plasma membrane part (65 genes), endoplasmic reticulum (37 genes), Golgi apparatus (33 genes), organelle membrane (28 genes), vesicle (25 genes), cytoplasmic vesicle (24 genes), membrane-bounded vesicle (22 genes), cell projection (21 genes), cytoplasmic membrane-bounded vesicle and endomembrane system (21 genes), vacuole and internal side of plasma membrane (17 genes), lysosome, lytic vacuole (15 genes), proteinaceous extracellular matrix (16 genes), extracellular matrix (16 genes), intracellular organelle lumen (15 genes), and organelle lumen (15 genes). However, GO terms, plasma membrane (102 genes), cytosol (14 genes), anchored to membrane (12 genes), and perinuclear region of cytoplasm (10 genes), were enriched with *p* value ≤ 0.05 (Figure 2, Additional File S1, Sheet 2). The cellular component GO terms assigned to retinal transcriptome of aged human and mice also showed the GO terms like plasma membrane in human [25] and rat [26].

#### 3.3.3. GO: Molecular Function

**Figure 3:**
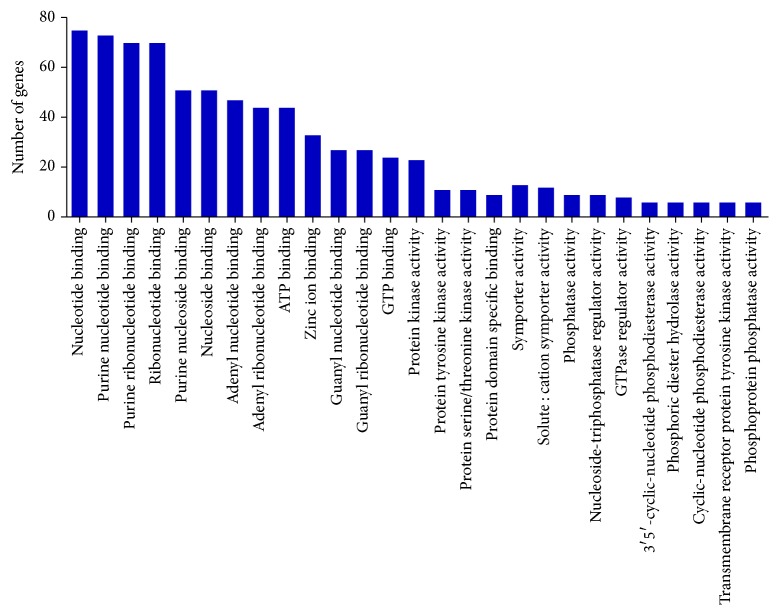
Functional annotation of expressed genes of retinal tissue in GO term: molecular function.

The enrichment of expressed genes in cellular component was observed for 293 genes which ranged between 75 and 6 genes in 27 GO terms, namely, nucleotide binding (75 genes), purine nucleotide binding (73 genes), purine ribonucleotide binding, ribonucleotide binding (70 genes), purine nucleoside binding (51 genes), nucleoside binding (51 genes), adenyl nucleotide binding (47 genes), adenyl ribonucleotide binding (44 genes), ATP binding (44 genes), zinc ion binding (33 genes), guanyl nucleotide binding (27 genes), guanyl ribonucleotide binding (27 genes), GTP binding (24 genes), protein kinase activity (23 genes), protein tyrosine kinase activity (11 genes), protein serine/threonine kinase activity (11 genes), and protein domain specific binding (9 genes). In addition, symporter activity (13 genes), solute : cation symporter activity (12 genes), phosphatase activity (9 genes), and nucleoside-triphosphatase regulator activity (9 genes) were enriched with *p* value ≤ 0.01 (Figure 3, Additional File S1, Sheet 3). This is consistent with retinal transcriptome reports on aged human [25] and rat [26].

#### 3.3.4. Retina-Specific Gene Expression Profiles

In an attempt to determine the retina associated expression of candidate genes, expression of 160 genes out of 221 genes (based on RetNet (https://sph.uth.edu/retnet/home.htm) gene list, January 2014) was detected. Among that, candidate genes like RHO, PDC, RPGR, CNGB1, SLC1A2, SLC1A3, SLC24A1, PRCD, PDE6G, PDE6A, PDE6B, PDE6H, and PDE6C were detected (expression values were presented in Additional File S1, Sheet 4). The GO analysis revealed that all of these genes were enriched in the categories of sensory perception of light stimulus, visual perception, response to radiation, and response to light stimulus (Table 3).

### 3.4. Assembly of Transcriptome and Comparative Analysis

**Figure 4:**
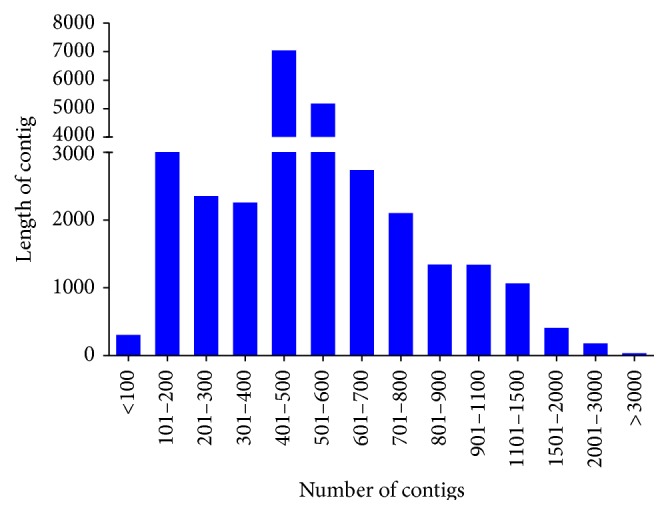
Distribution of contigs length in canine transcriptome assembly.

The reads were assembled using CAP3 which generated a total of 29,683 contigs with N50 of 619 and mean length of 560.9 bases (Table 4). Most of the contigs were in the range between 400 bp and 500 bp (Figure 4). The assembled sequences were compared against the NCBI nr database (ftp://ftp.ncbi.nlm.nih.gov/blast/db) using BLASTx (*e*-value 1*∗*10^−6^). Of the 29,683 assembled sequences, 12,498 (42.10%) contigs had significant hits corresponding to a single or more than one unique accession number to the nr database. The sequences hits with nr database using BLASTx were 42.10% with known functions.

### 3.5. Full-Length cDNA Prediction

Full-length cDNAs are important resources for many genetic and genomic researchers and useful to predict protein sequences [27]. All contigs were analysed by online tool Target Identifier to identify potential full-length cDNAs with complete open reading frame (ORF) in assembled transcriptome of canine retina. A total of 3,827 full-length, 914 short full-length, 331 ambiguous, 2,932 partial (5′-sequenced partial), and 2,913 3′-sequenced partial sequences were identified with a cutoff *e*-value of 10^−5^ (Figure 6).

### 3.6. Open Reading Frame (ORF) Identification

Open reading frame identification in RNA-Seq is important in gene prediction and identification of candidate protein coding regions [27]. In the present study, out of 29,683 total assembled contigs of canine retina transcriptome, 29,418 (99%) open reading frames (ORFs) were identified with an average length of 285 bp ranging from 50 bp to 5,696 bp (Figure 7). The remaining 265 contigs contained no ORFs, which indicates that these contigs were noncoding or originated from untranslated regions (UTRs).

### 3.7. Analysis of SSR/Microsatellites

Out of 29,683 contigs, a total of 2,470 SSRs/microsatellites were identified in 2,298 contigs, including dinucleotide (1,582) (68.84%), trinucleotide (592) (25.76%), tetranucleotide (216) (9.40%), pentanucleotide (58) (2.52%), and hexanucleotide (22) (0.96%) (Figure 8). The most predominant types among the dinucleotide repeat motifs were (AG/GA)_*n*_, (CT/TC)_*n*_, (AC/CA)_*n*_, (GT/TG)_*n*_, (AT/TA)_*n*_, and (CG/GC)_*n*_, with frequencies (in percentage) of 27.88, 23.45, 18.14, 16.56, 11.95, and 1.71, respectively. In the 20 types of trinucleotide repeats, GGC (15.20%) was the major common motif, followed by GCC (12.16%), TCC (9.46%), GGA (8.78%), GTC (6.42%), and TTC (5.91%) repeat motifs (Figure 8). These SSRs markers offer a valuable resource for further genetic investigations.

### 3.8. KEGG Pathway Annotation

In order to identify the active biological pathways in canine tissue, the assembled contigs were used to obtain the enzyme commission (EC) against the Kyoto Encyclopaedia of Genes and Genomes (KEGG) database. A total of 3,782 contigs were assigned to 3,570 enzyme commission (EC) numbers (Table 5). The assignments of contigs with metabolism pathways were predominant. Enzymes involved in retinal tissue metabolism were further classified into 12 subcategories. There were considerably a higher number of enzymes participating in the metabolism of carbohydrate (238 pathways), lipid (167 pathways), amino acid (164 pathways), and glycan biosynthesis and metabolism (115 pathways) (Table 5), indicating enormous tissue activities. Assignment to EC number of human disease was 832 (23.31%), which included cancers, infectious diseases, neurodegenerative disease, and other categories. Organismal system EC number assignment was 705 (19.75%), which included endocrine system, nervous system, and immune system. Environmental Information Processing enzymes were 340 (9.52%) which included signal transduction and signalling molecule interaction. Cellular Processes were 276 (7.73%) which included transport and catabolism, cell communication, cell growth and death, and cell motility. Genetic Information Processing was 204 (5.71%) which included folding sorting and degradation, translation, replication and repair, and transcription (Table 5).

#### 3.8.1. Possible Genes Related to Melanogenesis, Phototransduction, and Retinol Metabolism

The transcriptome of canine tissue was primarily examined to identify a wide range of candidate genes that might be functionally associated with melanogenesis, phototransduction, and retinol metabolism (Additional File 2, Table  S1 a). The present study indicated that 33 contigs were associated with melanogenesis pathways including adenylate cyclase with the EC number EC:4.6.1.1 encoded by five contigs, while protein kinase A (EC:2.7.11.11), CREB-binding protein (EC:2.3.1.48), RAF protooncogene serine/threonine-protein kinase (EC:2.7.11.1), and mitogen-activated protein kinase kinase 1 (EC:2.7.12.2) were encoded by two contigs. However, mitogen-activated protein kinase 1/3 (EC:2.7.12.2), dopachrome tautomerase (EC:5.3.3.12), phosphatidylinositol phospholipase C, beta (EC:3.1.4.11), calcium/calmodulin-dependent protein kinase (CaMkinase) II (EC:2.7.11.17), and classical protein kinase C (EC:2.7.11.13) were encoded by one contig (Additional File 2). Contigs associated with phototransduction pathways were 15, with rhodopsin kinase (EC:2.7.11.14, 1 contig) and rod cGMP-specific 3′,5′-cyclic phosphodiesterase (EC:3.1.4.35, 2 contigs) (Additional File 2, Table  S1 b). Enzymes involved in retinol metabolism were also encoded by the canine retinal tissue contigs (11 contigs). In the retinol metabolism, main enzymes detected were, namely, alcohol dehydrogenase (EC:1.1.1.1, 1 contig), retinol dehydrogenase (EC:1.1.1, 4 contigs), retinoid isomer hydrolase (EC:3.1.1.64, 3 contigs), and retinal dehydrogenase (EC:1.2.1.36, 1 contig) (Additional File 2, Table  S1 c).

## 4. Discussion

We performed RNA-Seq of canine retina using 454 GS-FLX and Ion Torrent PGM. Mapping identified the expression of 10,360 genes out of 28,455 reference genes of canine genome. The expressed genes were utilized for the gene ontology analysis and functional annotation.

In the retinal transcriptome, 316 genes were found to be enriched into the 28 molecular function GO category (*p* value < 0.01). The enrichment of genes in GO terms, namely, sensory perception of light stimulus, visual perception, response to light stimulus, response to radiation, cell adhesion, and biological adhesion, was consistent with retinal transcriptome of aged human and rat [25, 26]. The enrichment of genes to these functional categories was further established by RetNet (https://sph.uth.edu/retnet/home.htm) database. These visual functions together serve as the series of events required for an organism to receive a stimulus, convert it to a molecular signal, and recognize and characterize the signal.

Cellular component refers to the place in the cell where a gene product is active. These terms reflect our understanding of eukaryotic cell structure. In the retinal transcriptome, 293 genes were found to be enriched into the 34 cellular component GO category (*p* value < 0.01). The cellular component GO terms assigned to retinal transcriptome of aged human and mice also showed the GO terms like plasma membrane in human [25] and rat [26]. Molecular function refers to the elemental activity or task performed, or potentially performed, by individual gene products. In the retinal transcriptome, 293 genes were found to be enriched into the 27 molecular function GO category (*p* value < 0.01).

**Figure 5:**
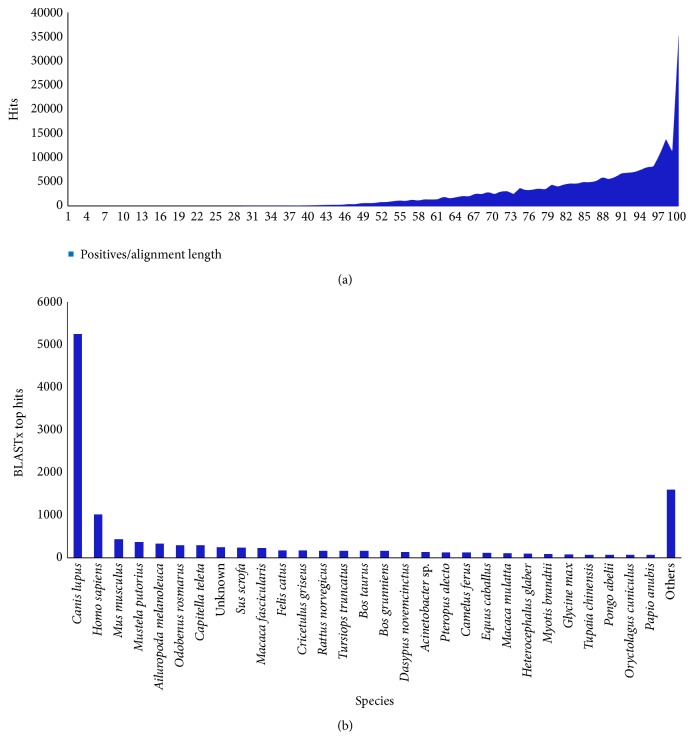
BLASTx hits distribution with *e*-value 10^−6^ against NCBI nr database and sequence similarity (a) and species distribution (b).

**Figure 6:**
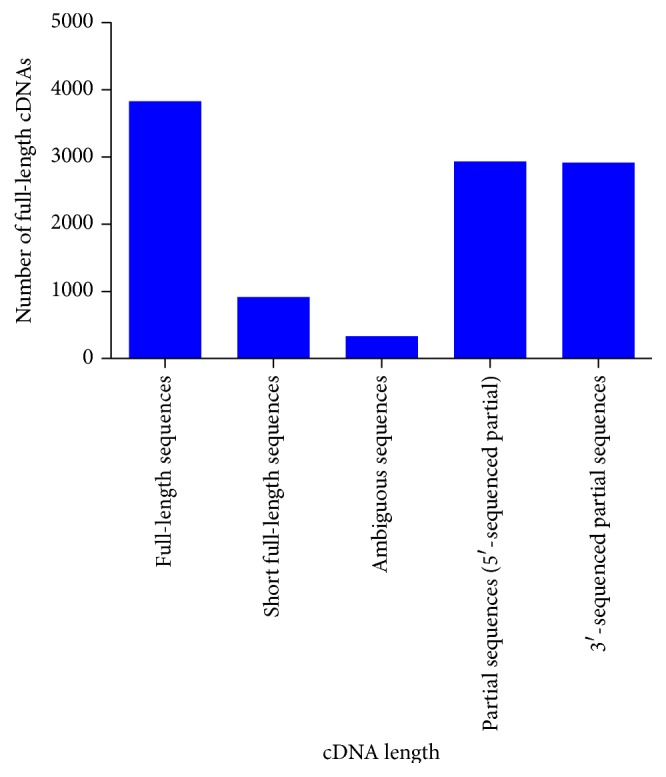
Length distribution of putative predicted full-length cDNA of assembled contigs of* C. lupus familiaris*.

**Figure 7:**
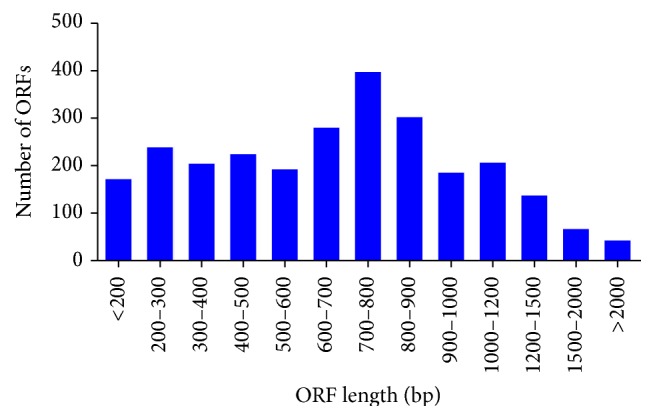
Open reading frame (ORF) length distribution of assembled contigs of* C. lupus familiaris*.

The BLASTx search of contigs revealed 42% of the hits matching* Canis lupus* whereas 8.12% matched* Homo sapiens*. Further *e*-value score and top hits distribution shows that maximum sequences were at the range of 90–100% similarity to reference. However, the poor annotation efficiency could be due to insufficient sequences in public databases for phylogenetically closely related species to date and limited sequence similarity of assembled contigs against NCBI nr database (Figure 5). Additionally, sequences without annotations may represent poorly conserved regions (e.g., untranslated regions (UTRs)) in* Canis lupus familiaris*. These values were higher than those in the comparable BLAST results from most other published studies using shotgun generated* de novo* transcriptomes [28–30].

Full-length cDNAs are the valuable source of information for many genetic and genomic researchers and can be useful to predict the protein sequences [27]. A total of 3,827 full-length cDNA sequences were identified which will serve as base for further cloning and gene expression analysis. A total of 29,418 (99%) ORFs were predicted from 29,683 contigs. These predicted ORFs indicate that most of the contigs have a protein coding sequence and derived from the exonic region of genes. The average length of predicted ORFs was 285 bp. The assembled transcriptome contigs can serve as a reference for cSNPs (Coding SNP) identification from transcriptome data for multiple canine breeds. ORF analysis would enable us to discriminate synonymous and nonsynonymous SNPs and to identify nonsense mutations in canines. Next-generation sequencing has identified ORF in* Anopheles funestus* [31] and plant species [32]. However, there are no reports of predicted ORFs identification in canine to date.

**Figure 8:**
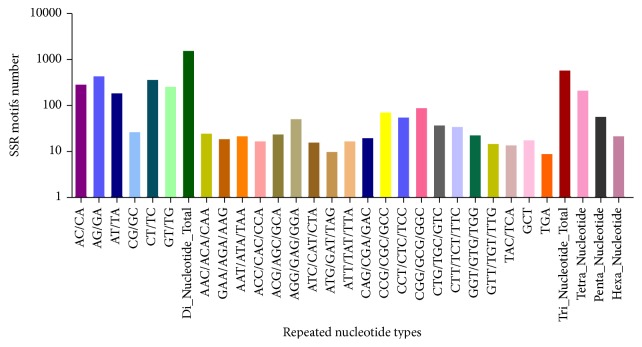
Distribution of simple sequence repeats (SSRs) among different nucleotide types found in the transcriptome of* C. lupus familiaris* assembled sequence.

The transcriptome sequencing provides an excellent source for mining and development of gene-associated markers [33, 34]. Microsatellites or SSRs are molecular markers that are widely distributed in a genome. They consist of repeated core sequences of 2–6 base pairs in length. SSRs have proven to be an efficient tool for performing QTL analysis, constructing genetic linkage, and evaluating the level of genetic variation in a species on account of high diversity, abundance, neutrality, and codominance of microsatellite markers [35, 36]. In the identified SSRs, the most dominant SSRs were dinucleotide repeats [(AG/GA)_*n*_, (CT/TC)_*n*_, (AC/CA)_*n*_, (GT/TG)_*n*_, (AT/TA)_*n*_, and (CG/GC)_*n*_] followed by trinucleotides [GGC, GCC (12.16%), TCC (9.46%), GGA (8.78%), GTC (6.42%), and TTC] (Figure 8). Unlike dog retina, the dinucleotide repeat of AC/GT type is the most abundant in Liaoning cashmere goat [37] compared to other vertebrates [38] but is different from plants [39]. Shotgun sequencing has identified numerous SSRs in plant species [40]. However, there are no reports of SSRs in dog retinal sample in India. We excluded mononucleotide SSRs in our analysis because of the common homopolymer errors that can occur in 454 GS-FLX and Ion Torrent sequencing data. These SSRs markers may offer a valuable resource for genetic variation study and further genetic investigations to be required in large dataset.

Vision is one of the most fascinating mechanisms of the interactions of a biological system and the process of phototransduction where the electromagnetic radiation is converted into biologically recognizable signals by the retinal photoreceptor cell. The phototransduction cascade of vertebrate serves as a benchmark system in signal transduction for a number of light stimuli including the remarkable ability of rod cells to respond reliably to single photon [41, 42]. In this study, we detected numerous contigs encoding EC number to phototransduction pathway. Strunnikova et al. [27] noted that several of the highly expressed signature genes encode proteins involved in visual cycle, melanogenesis, and cell adhesion in retinal pigment epithelium. The melanogenesis is essential for removal of toxic substances from the choroid and protects the retina from oxidative and chemical stress [43, 44]. The retinol metabolism (biosynthesis of Vitamin A) is essential for the life of all chordates. It has numerous important functions including a role in vision, maintenance of epithelial surfaces, and immune competence [27]. Multiple genes have been characterized to encode the components of this cycle and linked to many human retinal diseases [45].

## 5. Conclusion

In the present study, we have analyzed transcriptome data and identified >10000 expressed genes in canine retinal tissue. The enrichment of genes in GO terms, namely, sensory perception of light stimulus, visual perception, response to radiation, and response to light stimulus, suggested the abundance of genes specifically present in retinal tissue and involved in vision related processes. Several highly expressed genes encoding proteins involved in melanogenesis, phototransduction, and retinol metabolism were identified in the retina. Moreover, a large number of cDNA SSRs were predicted which can be used for subsequent marker development, genetic linkage, and QTL analysis. Overall, the canine retina transcriptome represents a valuable resource for future functional and comparative genomic studies for effective way to treat vision related problem of this globally vulnerable species.

## Supplementary Material

The Additional File S1 contains the generated data after enrichment of genes in GO category. The sheet 4 contains the expression value of expressed genes which were involved in retina specific gene expression profiles. The Additional File S2 contains KEGG orthology assignment of Melanogenesis, phototransduction and retinol metabolism.

## Figures and Tables

**Table 1:** Number and proportion of reads mapped to reference genome. Mapping statistics were carried out using CLC Genomics with *Canis lupus familiaris* genome build version CanFam3.1 as a reference (NCBI).

	Canine retina (expressed genes)
Total reads	800,154
Counted fragments	226,684
Uniquely mapped	222,296
Mapped unspecifically	4,388
Uncounted fragments	573,470
Number of genes	10,360
Number of genes in reference	28,455

**Table 2:** Distribution of mapped reads to different transcript types and gene regions with reference *Canis lupus familiaris* genome build version CanFam3.1.

	Uniquely mapped	Nonspecifically mapped	Mapped reads	
	Number of reads	Number of reads	Number of reads	%
Total exon reads	75,883	1,305	77,188	34.05
Exon-exon reads	32,385	768	33,153	14.63
Exon-intron reads	6,210	35	6,245	2.75
Total intron reads	107,818	2,280	110,098	48.57
Total gene reads	222,296	4,388	226,684	100.00

**Table 3:** GO terms biological process enrichment of expressed genes in retinal tissue.

GO ID	GO terms	Count	*p* value	Genes
GO:0050953	Sensory perception of light stimulus	17	0.006	GNAT1, RP1, RPGR, RPE65, RCVRN, PRPH2, PDE6G, CNGA1, GUCY2D, SAG, PDE6A, PDE6B, PDE6D, PRCD, PDC, CLN5, and RHO

GO:0007601	Visual perception	17	0.006	GNAT1, RP1, RPGR, RPE65, RCVRN, PRPH2, PDE6G, CNGA1, GUCY2D, SAG, PDE6A, PDE6B, PDE6D, PRCD, PDC, CLN5, and RHO

GO:0009314	Response to radiation	11	0.011	GNAT1, GNGT1, SLC1A2, SLC1A3, UACA, CASP9, BAX, RCVRN, BCL2L1, SNAI2, and RHO

GO:0009416	Response to light stimulus	9	0.017	GNAT1, GNGT1, SLC1A2, SLC1A3, UACA, CASP9, BAX, RCVRN, and RHO

**Table 4:** Summary statistics of *C. lupus familiaris* reads and assembled contigs.

Features	Values
Clean reads for assembly	800,154
N50 length	619
N75 length	485
N90 length	384
Contig number	29,683
Contig bases	16,649,067
Maximum contig length	6,416
Minimum contig length	42
Mean length of cleaned reads	560.9
Mode length of cleaned reads	510
Median length of cleaned reads	501
Number of reads per contig	27.95

**Table 5:** KEGG biochemical mappings for *C. lupus familiaris:* enzyme commission assignment of assembled transcripts.

KEGG pathway	EC count (unique transcripts)
Metabolism	**1213**
Overview	135
Carbohydrate metabolism	238
Energy metabolism	96
Lipid metabolism	176
Nucleotide metabolism	79
Amino acid metabolism	164
Metabolism of other amino acids	53
Glycan biosynthesis and metabolism	115
Metabolism of cofactors and vitamins	67
Metabolism of terpenoids and polyketides	19
Biosynthesis of other secondary metabolites	17
Xenobiotics biodegradation and metabolism	54
Genetic Information Processing	**204**
Transcription	21
Translation	54
Folding, sorting, and degradation	97
Replication and repair	32
Environmental Information Processing	**340**
Signal transduction	330
Signaling molecules and interaction	10
Cellular Processes	**276**
Transport and catabolism	115
Cell motility	18
Cell growth and death	70
Cell communication	73
Organismal Systems	**705**
Immune system	151
Endocrine system	196
Circulatory system	26
Digestive system	67
Excretory system	37
Nervous system	163
Sensory system	15
Development	30
Environmental adaptation	20
Human Diseases	**832**
Cancers	320
Immune diseases	23
Neurodegenerative diseases	114
Substance dependence	42
Cardiovascular diseases	12
Endocrine and metabolic diseases	24
Infectious diseases	297
Total	**3570**

## Abbreviations

RPE: Retinal pigment epithelium
IRD: Inherited retinal degeneration
RPKM: Reads per kilobase per million mapped reads
EST: Expressed Sequence Tag
GO: Gene ontology
CAP3: Contig assembly program-3
ORF: Open reading frame
KEGG: Kyoto Encyclopedia of Genes and Genomes
nr: Nonredundant
SSR: Simple sequence repeat.
