# Supplementary material for: De Novo Assembly and Transcriptome Characterization of Canine Retina Using High-Throughput Sequencing
Source: Genet Res Int. 2015 Dec 16;2015:638679. doi: 10.1155/2015/638679 (PMC4695645; doi:10.1155/2015/638679)
Supplement: Supplementary file 1 — The Additional File S1 contains the generated data after enrichment of genes in GO category. The sheet 4 contains the expression value of expressed genes which were involved in retina specific gene expression profiles. The Additional File S2 contains KEGG orthology assignment of Melanogenesis, phototransduction and retinol metabolism. [file 638679.f1.zip › 638679 Additional file S2.docx]

Table S1 a: Assignment of Melanogenesis KEGG orthology (KO: 04196) in retinal Transcriptome

| Contigs | Genes | Enzyme/Protein | EC Code | |
| --- | --- | --- | --- | --- |
| Contig1979 | GNAS | guanine nucleotide-binding protein G(s) subunit alpha | | - |
| Contig20120 | ADCY1 | adenylate cyclase 1 | EC:4.6.1.1 | |
| Contig16159 | ADCY2 | adenylate cyclase 2 | EC:4.6.1.1 | |
| Contig17719 | ADCY5 | adenylate cyclase 5 | EC:4.6.1.1 | |
| Contig12588 | ADCY6 | adenylate cyclase 6 | EC:4.6.1.1 | |
| Contig13394 | ADCY9 | adenylate cyclase 9 | EC:4.6.1.1 | |
| Contig14816 | PKA | protein kinase A | EC:2.7.11.11 | |
| Contig24557 | PKA | protein kinase A | EC:2.7.11.11 | |
| Contig5553 | CREB3 | cyclic AMP-responsive element-binding protein 3 | - | |
| Contig13051 | CREB3 | cyclic AMP-responsive element-binding protein 3 | - | |
| Contig3537 | EP300, CREBBP, KAT3 | E1A/CREB-binding protein | EC:2.3.1.48 | |
| Contig5651 | EP300, CREBBP, KAT3 | E1A/CREB-binding protein | EC:2.3.1.48 | |
| Contig11353 | MITF | microphthalmia-associated transcription factor | - | |
| Contig7507 | FZD1_7, fz | frizzled 1/7 | - | |
| Contig2891 | FZD6 | frizzled 6 | - | |
| Contig23663 | GNAQ | guanine nucleotide-binding protein G(q) subunit alpha | - | |
| Contig8272 | DVL | segment polarity protein dishevelled | - | |
| Contig19341 | GSK3B | glycogen synthase kinase 3 beta | EC:2.7.11.26 | |
| Contig20916 | CTNNB1 | catenin beta 1 | - | |
| Contig13475 | NRAS | GTPase NRas | - | |
| Contig3563 | RAF1 | RAF proto-oncogene serine/threonine-protein kinase | EC:2.7.11.1 | |
| Contig16322 | RAF1 | RAF proto-oncogene serine/threonine-protein kinase | EC:2.7.11.1 | |
| Contig5629 | MAP2K1, MEK1 | mitogen-activated protein kinase kinase 1 | EC:2.7.12.2 | |
| Contig24293 | MAP2K2, MEK2 | mitogen-activated protein kinase kinase 2 | EC:2.7.12.2 | |
| Contig20776 | MAPK1_3 | mitogen-activated protein kinase 1/3 | EC:2.7.11.24 | |
| Contig7811 | DCT | dopachrome tautomerase | EC:5.3.3.12 | |
| Contig4438 | EDNRB | endothelin receptor type B | - | |
| Contig12660 | GNAI | guanine nucleotide-binding protein G(i) subunit alpha | - | |
| Contig7341 | PLCB | phosphatidylinositol phospholipase C, beta | EC:3.1.4.11 | |
| Contig5424 | CALM | calmodulin | - | |
| Contig7350 | CALM | calmodulin | - | |
| Contig16986 | CAMK2 | calcium/calmodulin-dependent protein kinase (CaMkinase) II | EC:2.7.11.17 | |
| Contig16125 | CPKC | classical protein kinase C | EC:2.7.11.13 | |

Table S1 b: Assignment of phototrasduction KEGG orthology (KO: 04744) in retinal Transcriptome.

| Contigs | Genes | Enzyme/Protein | EC Code |
| --- | --- | --- | --- |
| Contig21303 | RHO, OPN2 | rhodopsin | - |
| Contig20736 | GRK1_7 | rhodopsin kinase | EC:2.7.11.14 |
| Contig13685 | RCVRN | recoverin | - |
| Contig24240 | GNAT | guanine nucleotide-binding protein G(t) subunit alpha | - |
| Contig14748 | GNB1 | guanine nucleotide-binding protein G(I)/G(S)/G(T)subunit beta-1 | - |
| Contig22520 | RGS9 | regulator of G-protein signaling 9 | - |
| Contig16302 | PDE6A | rod cGMP-specific 3',5'-cyclic phosphodiesterasesubunit alpha | EC:3.1.4.35 |
| Contig19083 | PDE6B | rod cGMP-specific 3',5'-cyclic phosphodiesterasesubunit beta | EC:3.1.4.35 |
| Contig19137 | PDE6G | retinal rod rhodopsin-sensitive cGMP 3',5'-cyclic phosphodiesterase subunit gamma | - |
| Contig18456 | GUCA1 | guanylate cyclase activator 1 | - |
| Contig20625 | GUCA1 | guanylate cyclase activator 1 | - |
| Contig15308 | SLC24A1, NCKX1 | solute carrier family 24 (sodium/potass ium/calcium exchanger), member 1 | - |
| Contig5424 | CALM | calmodulin | - |
| Contig7350 | CALM | calmodulin | - |
| Contig17985 | CNGA1 | cyclic nucleotide gated channel alpha 1 | - |

Table S1 c: Assignment of retinol metabolism KEGG orthology (KO: 00830) in retinal transcriptome.

| Contigs | Genes | Enzyme | EC Code |
| --- | --- | --- | --- |
| Contig20089 | ADH4 | alcohol dehydrogenase 4 | EC:1.1.1.1 |
| Contig19896 | frmA, ADH5, adhC | S-(hydroxymethyl)glutathione dehydrogenase / alcohol dehydrogenase | EC:1.1.1.284 1.1.1.1 |
| Contig16048 | DHRS4 | dehydrogenase/reductase SDR family member 4 | EC:1.1 |
| Contig23455 | RDH8 | retinol dehydrogenase 8 | EC:1.1.1 |
| Contig4985 | RDH10 | retinol dehydrogenase 10 | EC:1.1.1 |
| Contig24167 | RDH12 | retinol dehydrogenase 12 | EC:1.1.1 |
| Contig13877 | RPE65 | retinoid isomerohydrolase | EC:3.1.1.64 |
| Contig19292 | RPE65 | retinoid isomerohydrolase | EC:3.1.1.64 |
| Contig20815 | RPE65 | retinoid isomerohydrolase | EC:3.1.1.64 |
| Contig16037 | RDH5 | retinol dehydrogenase | EC:1.1.1 |
| Contig1024 | E1.2.1.36 | retinal dehydrogenase | EC:1.2.1.36 |
